# Does previous history of cancer or atypia predict histologic upgrade for pure intraductal papillomas diagnosed via core biopsy? A study of 490 cases at a single institution

**DOI:** 10.1002/cnr2.1481

**Published:** 2021-11-02

**Authors:** J. Jaime Alberty‐Oller, Sylvia Reyes, Erin Moshier, Meng Ru, Sarah Weltz, Antonio Santos, Kereeti Pisapati, Elisa Port, Shabnam Jaffer

**Affiliations:** ^1^ Mount Sinai Hospital Dubin Breast Center of the Tisch Cancer Institute, Icahn School of Medicine at Mount Sinai New York New York USA

**Keywords:** breast cancer, core biopsy, intraductal papilloma

## Abstract

**Background:**

Management of pure intraductal papillomas (IDP) without atypia diagnosed on core needle biopsy (CNB) remains controversial given highly variable rates of upgrade in the literature.

**Aim:**

We sought to identify clinical and histologic factors that predict upgrade to atypia or malignancy in a large population.

**Methods and results:**

A retrospective review was performed of all cases of pure IDP diagnosed on CNB and then surgically excised at a single institution from 2008 to 2018. Clinical, radiologic, and pathologic factors were compared in the no upgrade, upgrade to atypia, or upgrade to cancer groups. Univariate analysis was performed comparing no upgrade and upgrade to cancer or atypia.

Four hundred and thirty nine patients were identified with a total of 490 IDP and a median age of 50 years (range 16–85). Of these patients, 54 (12.3%) were upgraded to atypia after surgical excision and five (1.1%) were upgraded to cancer. The presence of multiple papillomas in a single patient was a significant predictor of upgrade to cancer or atypia (*p* < .01), as well as age over ≥55 years (*p* < .01) and a prior history of cancer (*p* < .01). No other clinical, radiologic and histologic factors were found to be significant predictors of upgrade. 40/439 (9.1%) patients in the total cohort had prior history of cancer, and of these, 2/40 (5%) were found to have a new cancer after excision.

**Conclusions:**

In patients with pure IDP on CNB, the upgrade rate to malignancy was 1.1%, while 12.3% were upgraded to atypia. The clinical significance of identifying atypia in a papilloma is unknown, especially in a patient with a prior history of atypia or cancer. However, the majority of patients who were upgraded to either atypia or cancer had no prior history of high‐risk or malignant breast disease and are therefore considered true clinical upgrades. As such excision for IDP should be considered.

## 1 INTRODUCTION

Intraductal papillomas (IDP) are benign papillary lesions of the breast with fibrovascular cores comprised of ductal and myoepithelial cells.^1^, ^2^ General consensus exists that IDP with atypia should be surgically excised, as these lesions have unacceptably high rates of upgrade when undergoing observation as opposed to excision, reported to be anywhere from 6.8% to 38.1%.^3^, ^4^, ^5^, ^6^, ^7^ Management of pure IDP—those without atypia or malignancy identified after core needle biopsy (CNB)—remains controversial even today, due to highly variable rates of upgrade in the literature.^8^, ^9^, ^10^, ^11^, ^12^ Previous studies have failed to consistently identify clinical, radiologic, and pathologic factors associated with possibility of upgrade, although some have suggested increased likelihood related to IDP size, multiplicity, presence of microcalcifications on imaging, nipple discharge on presentation, peripheral rather than central location, and patient older age.^13^, ^14^ Additionally, there is a paucity of data on how a patient's history of atypical breast disease or breast cancer (BC) may affect possibility of upgrade.

We sought to identify clinical, histologic and radiologic factors that predict upgrade of pure IDP to either atypia or malignancy, and to investigate the relevance of prior history of breast disease as it relates to the upgrade of these lesions on surgical excision after CNB.

## 2 METHODS

**FIGURE 1:**
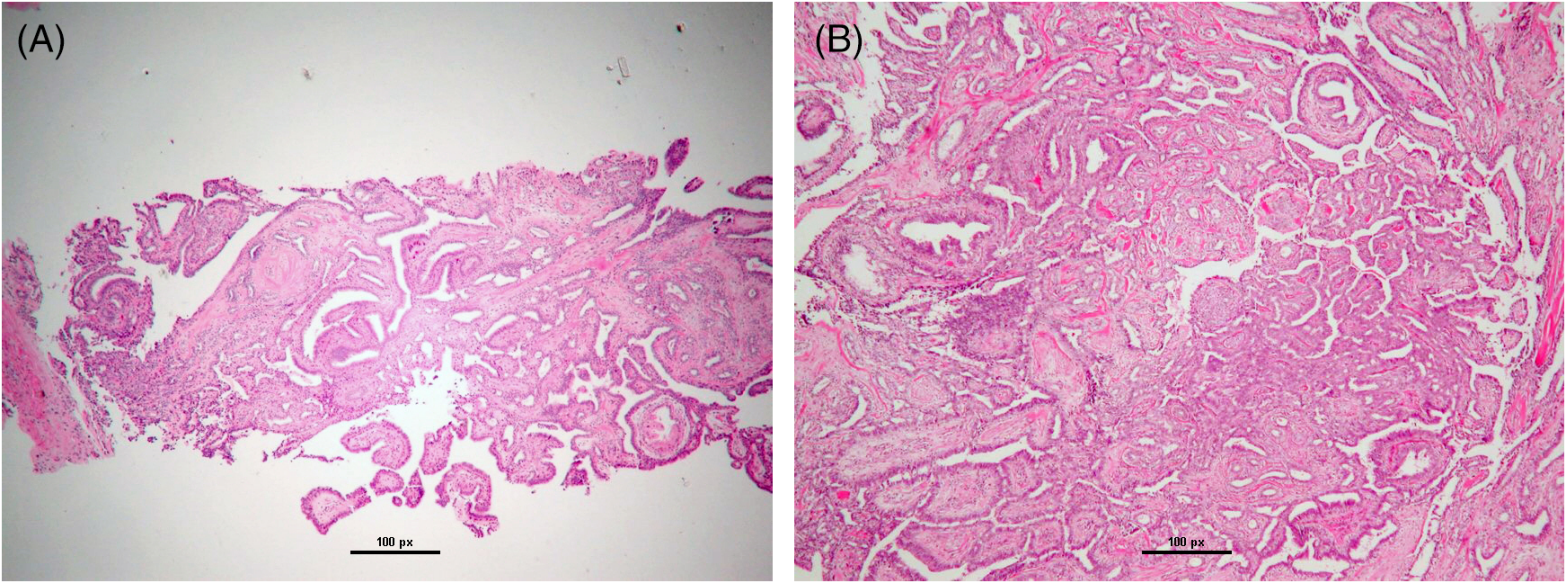
On the left (A), core needle biopsy showing arborizing papillary proliferation diagnostic of intraductal papilloma. On the right (B), surgical excision of needle biopsy from A, showing residual benign intraductal papilloma

**FIGURE 2:**
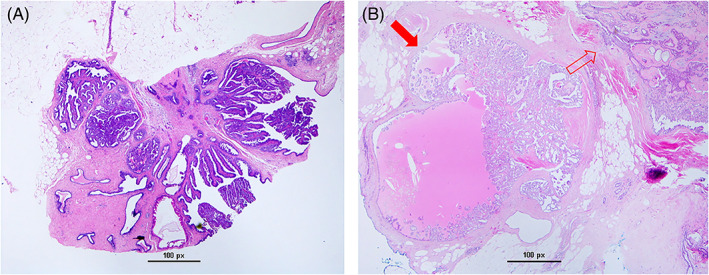
On the left (A), core needle biopsy showing arborizing papillary proliferation diagnostic of intraductal papilloma. On the right (B), surgical excision of needle biopsy from A: residual benign intraductal papilloma (unfilled arrow) and adjacent ductal carcinoma in situ (DCIS), highlighted by filled arrow

In this institutional review board (IRB) approved study, we retrospectively queried the pathology database at our institution for all cases of pure IDP diagnosed on CNB and subsequently surgically excised from 2008 to 2018. Core needle biopsies were performed by radiologists under stereotactic guidance, ultrasound guidance or magnetic resonance imaging (MRI) guidance. Pathology from core biopsy and surgical excision were reviewed by dedicated breast pathologists at our academic institution at the time of the procedure. Figure 1 is a representative microphotography of a pure IDP at time of biopsy and surgical excision (no upgrade), while Figure 2 highlights a case where upgrade from pure IDP to ductal carcinoma in‐situ (DCIS) was seen after surgical excision. We excluded cases of pathologic atypia or malignancy found on CNB at the site of IDP. After exclusions, we identified a total of 490 cases of pure IDP discovered on biopsies performed in 439 patients, as several patients had multiple sampled lesions.

The electronic medical records of these 439 patients were then reviewed, and the following information was recorded: patient demographics, associated physical findings and/or presenting complaint, prior history of breast disease including papillary lesions, high risk or atypical lesions and carcinoma of the breast, imaging modality used in the diagnostic evaluation, radiologic factors including size and location of lesion, biopsy gauge size, and pathologies from core biopsies and subsequent surgical excisions. On surgical excision, pathologic upgrades were separated into two groups: (1) high‐risk lesions (HRLs), defined as any atypia including atypical ductal hyperplasia (ADH) or lobular neoplasia (LN) and (2) carcinoma, including both DCIS and invasive disease. Information from the electronic medical records was then compared in the no upgrade, upgrade to HRL and upgrade to carcinoma groups, and quantitatively analyzed to determine which characteristics would prove statistically significant for predicting upgrade.

Patient and papilloma related characteristics were summarized overall and by upgrade status. Continuous variables were reported as median (range: min–max) and nominal variables were reported as *N* (%). Univariable comparisons were made with modified Poisson regression models using a robust error variance, introduced by Zou, implemented with SAS's Proc Genmod.^15^ Multivariable adjusted prevalence ratios and corresponding 95% confidence intervals were estimated using the modified Poisson regression models. A backward elimination process was used to select variables in the final adjusted models. All statistical analyses described above were performed using SAS Version 9.4 (SAS Institute, Cary, NC). Hypothesis testing was two‐sided and conducted at the 5% level of significance.

## 3 RESULTS

Our study identified 490 distinct IDP in 439 patients in the overall cohort. Patient demographics and presenting symptoms are summarized in Table 1. Median age was 50 years (range 16–85), with 168 (38%) patients being 55 years of age or over. The majority of cases (72%) were identified as part of routine imaging studies, whether yearly screening or short term follow up, while 10% presented with nipple discharge symptoms and 18% presented with complaints of a palpable mass. Most patients (82%) did not have any prior history of high risk or malignant disease, while 40 (9%) had a history of prior cancer and 16 (4%) had a history of previously biopsied atypia or other HRL. Twenty patients (5%) had undergone a prior biopsy yielding pure IDP.

**TABLE 1:** Patient characteristics, *N* = 439

Characteristic	Overall *N* = 439	Overall upgrade			Malignant upgrade		
		No *N* = 380	Yes *N* = 59	*p*‐Value	No *N* = 434	Yes *N* = 5	*p*‐Value
*Age, years*							
Median [min–max]	50 [16–85]	49 [16–85]	57 [26–81]	.0045^a^	50 [16–85]	54 [36–73]	.3527
<55, n (%)	271 (62%)	245 (64%)	26 (44%)	.0032^a^	268 (62%)	3 (60%)	.9362
≥55, n (%)	168 (38%)	135 (36%)	33 (56%)		166 (38%)	2 (40%)	
*Number of papillomas*							
Median [min–max]	1 [1–5]	1 [1–4]	1 [1–5]	.0010^a^	1 [1–5]	1 [1–4]	.0287^a^
Single, *n* (%)	398 (91%)	348 (92%)	50 (85%)	.0838	394 (91%)	4 (80%)	.4227
Multiple, *n* (%)	41 (9%)	32 (8%)	9 (15%)		40 (9%)	1 (20%)	
*Bilateral, n (%)*							
Unilateral	419 (95%)	363 (96%)	56 (95%)	.8328	415 (96%)	4 (80%)	.1303
Bilateral	20 (5%)	17 (4%)	3 (5%)		19 (4%)	1 (20%)	
*Presenting symptom*,^*b*^ *n (%)*							
Nipple discharge	42 (10%)	40 (11%)	2 (3%)	.1039	42 (10%)	0 (0%)	NE
Palpable mass	76 (18%)	65 (18%)	11 (19%)	.8690	74 (18%)	2 (40%)	.2159
Screening	257 (60%)	220 (60%)	37 (63%)	.7050	256 (70%)	1 (20%)	.1038
Follow‐up	49 (12%)	42 (11%)	7 (12%)	.9307	47 (11%)	2 (40%)	.0698
Other	33 (8%)	28 (8%)	5 (8%)	.8251	33 (8%)	0 (0%)	NE
*Missing*	*14 (3%)*	*14 (4%)*	*0 (0%)*		*14 (3%)*	*0 (0%)*	
*Personal history, n (%)*							
None	357 (82%)	317 (85%)	40 (68%)	.0012^a^	354 (83%)	3 (60%)	.2067
Cancer	40 (9%)	27 (7%)	13 (22%)	.0001^a^	38 (9%)	2 (40%)	.0363^a^
HRL	16 (4%)	12 (3%)	4 (7%)	.1561	16 (4%)	0 (0%)	NE
Papillary lesion	20 (5%)	18 (5%)	2 (3%)	.6366	20 (5%)	0 (0%)	NE
*Missing*	*6 (2%)*	*6 (2%)*	*0 (0%)*		*6 (1%)*	*0 (0%)*	

*Note*: Missing (%) is calculated out of the total 439 patients, Non‐missing (%) calculated out of total patients with available data overall upgrade: atypia, invasive or in situ carcinoma; malignant upgrade: invasive or in situ carcinoma.

Abbreviations: Max, maximum; Min, minimum; NE, not estimable due to cell counts of 0.

a Denotes statistical significance.

b Numbers do not sum to 439 and percentages do not sum to 100% due to some patients presenting with multiple symptoms.

IDP characteristics are summarized in Table 2, including breast laterality, size and location of lesion, imaging modality used for biopsy, and biopsy needle gauge size and number of passes during CNB. The majority of all IDPs sampled were located in the retroareolar breast (264, 54%), while 213 (44%) were peripherally located and 9 (2%) were located in the central area of the breast. In terms of imaging, most (409, 87%) were identified on ultrasound, while 43 (9%) were identified on mammography and 16 (3%) were identified as enhancing masses on MRI. Most of the sampled IDPs were 1 cm or under in size (312, 63.7%).

**TABLE 2:** Papilloma characteristics, *N* = 490

Characteristic	Overall *N* = 490	Overall upgrade			Malignant upgrade		
		No *N* = 426	Yes *N* = 64	*p*‐Value	No *N* = 484	Yes *N* = 6	*p*‐Value
*Laterality, n (%)*							
Left	268 (55%)	231 (54%)	37 (58%)	.3521	265 (55%)	3 (50%)	.3832
Right	222 (45%)	195 (46%)	27 (42%)		219 (45%)	3 (50%)	
*Location of papilloma, n (%)*							
Retroareolar	264 (54%)	234 (55%)	264 (54%)	.7272	262 (55%)	2 (33%)	NE
Peripheral	213 (44%)	180 (43%)	213 (44%)		209 (43%)	4 (67%)	
Central	9 (2%)	8 (2%)	9 (2%)		9 (2%)	0 (0%)	
*Missing*	*4 (1%)*	*4 (1%)*	*0 (0%)*		*4 (1%)*	*0 (0%)*	
*Imaging modality, n (%)*							
US	409 (87%)	361 (87%)	48 (87%)	.6346	405 (87%)	4 (80%)	NE
Stereo/mammo	43 (9%)	37 (9%)	6 (11%)		42 (9%)	1 (20%)	
MRI	16 (3%)	15 (4%)	1 (2%)		16 (3%)	0 (0%)	
*Missing*	*22 (5%)*	*13 (3%)*	*9 (14%)*		*21 (4%)*	*1 (17%)*	
*Papilloma size (mm)*							
Median [min–max]	7 [1–70]	7 [1–70]	7 [3–34]	.6625	7 [1–70]	11 [4–14]	.4607
1–5 mm, *n* (%)	119 (30%)	109 (31%)	10 (22%)	.0800	118 (30%)	1 (25%)	.5674
6–10 mm, *n* (%)	193 (48%)	166 (47%)	27 (59%)		192 (49%)	1 (25%)	
>10 mm, *n* (%)	86 (22%)	77 (22%)	9 (19%)		84 (21%)	2 (50%)	
*Missing, n (%)*	*92 (19%)*	*74 (17%)*	*18 (28%)*		*90 (19%)*	*2 (33%)*	
*Gauge size*							
Median [min–max]	12 [7–20]	12 [7–20]	12 [8–18]	.1306	12 [7–20]	13 [9–16]	.9233
≤12, *n* (%)	289 (62%)	250 (61%)	39 (67%)	.1150	286 (62%)	3 (50%)	NE
13, *n* (%)	7 (1%)	7 (2%)	0 (0%)		7 (2%)	0 (0%)	
14, *n* (%)	110 (24%)	95 (23%)	15 (26%)		108 (23%)	2 (33%)	
16, *n* (%)	23 (5%)	20 (5%)	3 (5%)		22 (5%)	1 (17%)	
≥18, *n* (%)	38 (8%)	37 (9%)	1 (2%)		38 (8%)	0 (0%)	
*Missing, n (%)*	*23 (5%)*	*17 (4%)*	*6 (9%)*		*23 (5%)*	*0 (0%)*	
*Number of cores sampled*							
Median [min–max]	3 [0–12]	3 [0–12]	3 [1–12]	.8996	3 [0–12]	4 [3–6]	.4828
≤2, *n* (%)	44 (12%)	38 (12%)	6 (13%)	.3954	44 (12.5%)	0 (0%)	NE
3, *n* (%)	146 (41%)	126 (40%)	20 (45%)		144 (41%)	2 (50%)	
4, *n* (%)	97 (27%)	89 (29%)	8 (18%)		97 (27%)	0 (0%)	
5, *n* (%)	45 (13%)	40 (13%)	5 (11%)		44 (12.5%)	1 (25%)	
≥6, *n* (%)	26 (7%)	20 (6%)	6 (13%)		25 (7%)	1 (25%)	
*Missing, n (%)*	*132 (27%)*	*113 (27%)*	*19 (30%)*		*130 (27%)*	*2 (33%)*	

*Note*: Missing (%) is calculated out of the total 490 papillomas, Non‐missing (%) calculated out of total papillomas with available data. Overall upgrade: atypia, invasive or in situ carcinoma; malignant upgrade: invasive or in situ carcinoma.

Abbreviations: Max, maximum; Min, minimum; NE, not estimable due to cell counts of 0.

**FIGURE 3:**
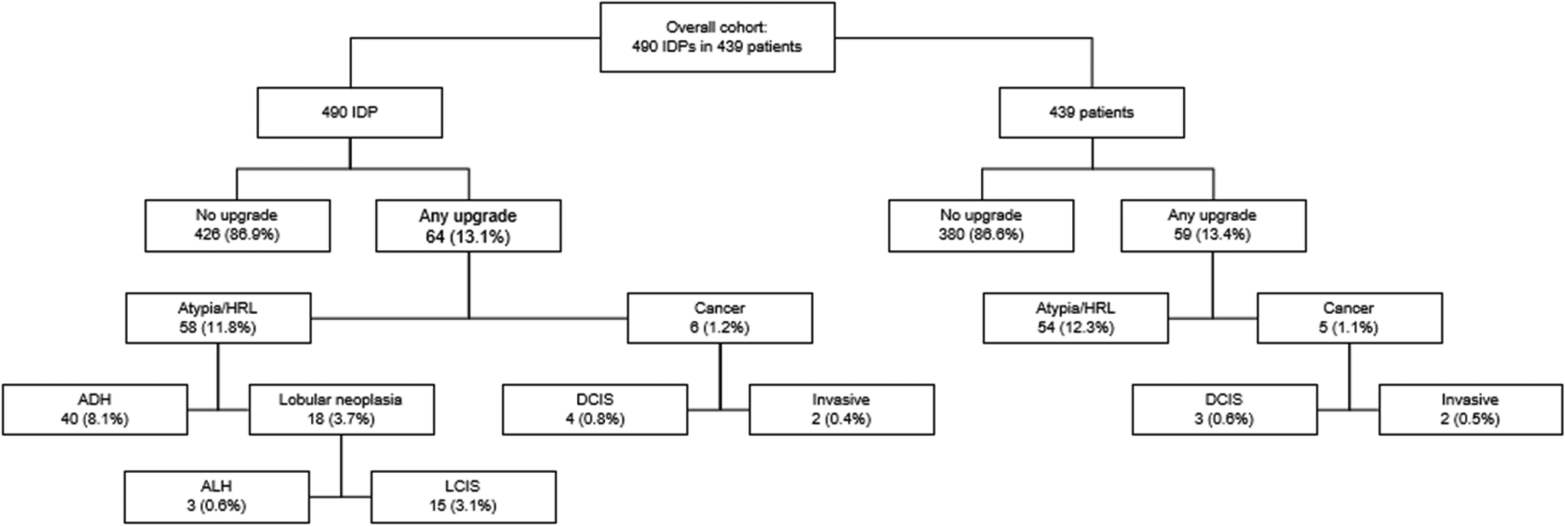
Case flow for upgrades within the entire study cohort. ADH, atypical ductal hyperplasia; ALH, atypical lobular hyperplasia; DCIS, ductal carcinoma in situ; HRL, high‐risk lesion; IDP, intraductal papilloma; LCIS, lobular carcinoma in situ

Figure 3 details the upgrade rates for the entire cohort. After surgical excision of the 490 IDPs, pathologic analysis identified no histologic upgrade in 426 (86.9%) cases; of these, we found residual pure IDP in 377 (76.9%) while 49 (10%) had no residual lesion. Overall, we identified an upgrade rate of 13.1% (64/490). Among these, 58 (11.8%) were upgraded to atypia/HRL while 6 (1.2%) were upgraded to carcinoma. A number of patients had multiple IDPs sampled, whether in the ipsilateral or the contralateral breast. When considering individual patients, 59/439 (13.4%) were upgraded: 54 (12.3%) to atypia/HRL and 5 (1.1%) to carcinoma, either DCIS or invasive disease.

**FIGURE 4:**
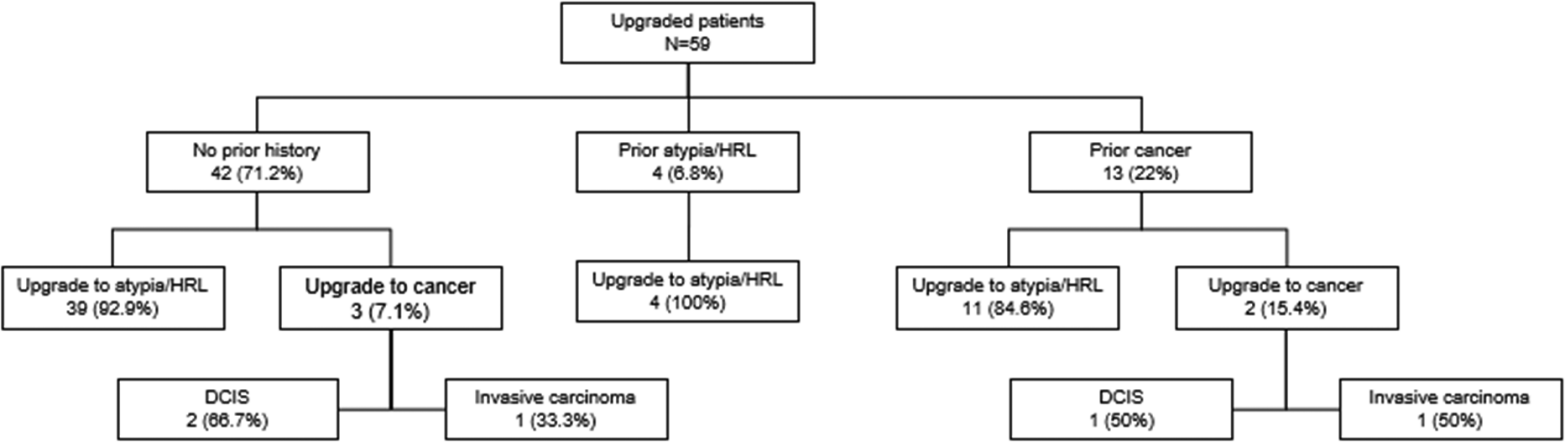
Upgraded patients by previous history of breast disease. DCIS, ductal carcinoma in situ; HRL, high‐risk lesion

When examining the 59 patients who were upgraded on surgical excision, the majority (42/59, 71.2%) had no previous history of high risk or malignant breast disease. Three (7.1%) of these patients without prior history were ultimately found to have DCIS or an invasive carcinoma on surgical excision. Only 4/59 (6.8%) patients had history of previous atypia—and all four upgraded to further atypia/HRL on excision. Thirteen (22.0%) patients had history of previously treated breast carcinoma, and of these, two were found to have a new breast cancer after surgical excision. Figure 4 outlines the upgraded patients by prior history of high‐risk or malignant breast disease.

Upon univariate analysis of all abstracted clinical, radiologic and histologic patient characteristics, only a few were found to be associated with upgrade on final excision (Table 1). The presence of multiple IDPs in a single patient, older age (≥55) at diagnosis, and a prior history of cancer were associated with any upgrade. Furthermore, only presence of multiple IDPs and personal history of cancer were significantly associated with malignant upgrade. No other clinical, radiologic and histologic factors were significantly associated with upgrade, including size of the IDP, size of the CNB, or number of core biopsy specimens taken.

On multivariable analysis, presence of each additional IDP in a single patient (PR 1.51 95% CI 1.17, 1.94), age 55 or over at diagnosis (PR 1.81 95% CI 1.09, 3.00) and a prior history of breast cancer (PR 2.14 95% CI 1.23, 3.71) remained independent factors of overall upgrade to either atypia or malignancy. Only presence of each additional IDP (PR 2.23 95% CI 1.23, 4.07) and a patient's personal history of breast cancer (PR 5.78 95% CI 1.21, 27.60]) were predictive factors of malignant upgrade to in situ or invasive carcinoma of IDPs (Table 3).

**TABLE 3:** Factors associated with overall upgrade (atypia, invasive, or in situ carcinoma) or malignant upgrade (invasive or in situ carcinoma) in multivariable modeling

Factor	Overall upgrade		Malignant upgrade	
	Multivariable PR [95% CI]	*p*‐Value	Multivariable PR [95% CI]	*p*‐Value
*Age, years*				
<55	*Reference*			
≥55	1.81 [1.09, 3.00]	.0209^a^		NS
*Number of papillomas*				
Per 1 papilloma increase	1.51 [1.17, 1.94]	.0014^a^	*2.23 [1.23, 4.07]*	.0085^a^
*Personal History of Breast Cancer*				
No cancer	*Reference*		*Reference*	
Cancer	2.14 [1.23, 3.71]	.0071^a^	5.78 [1.21, 27.60]	.0280^a^

*Note*: Interpretation: Patients 55 and older are 70% more likely than those below55 to have an excised papilloma upgrade to atypia/HRL/malignancy (PR: 1.70; 95% CI: [1.04, 2.77]). Patients with multiple papillomas are 79% more likely than patients with single papilloma to upgrade to atypia/HRL/malignancy (PR: 1.79; 95% CI: [1.06, 3.01]). Patients with a personal history of breast cancer are 125% more likely than those without history of breast cancer to have a papilloma upgrade to atypia/HRL/malignancy (PR: 2.25; 95% CI: [1.33, 3.83]).

Abbreviations: CI, confidence interval; NS, not significant; OR, odds ratio.

a Denotes statistical significance.

## 4 DISCUSSION

Although most studies generally recommend surgical excision for IDP with atypia found on CNB, appropriate management of pure IDP without atypia or malignancy remains unclear. The main worry espoused by those who recommend excision for all papillomas is a missed malignant disease diagnosis due to sampling error during a diagnostic CNB when complete surgical excision is not performed. In our study, for example, pathologist review of the CNB slides from the five patients that ultimately upgraded to carcinoma determined most of these were likely due to sampling error, while only one was felt to be a true misinterpretation of the core biopsy (atypical cells were present upon review).

For our study, and as several past studies have done before, we defined upgrade to include either atypical/HRLs or malignant histology on surgical excision, understanding that the identification of certain HRLs may alter clinical management including possible eligibility for chemoprophylaxis and more extensive high risk radiologic screening whether with whole breast sonography or MRI.^16^

Upgrade rates for pure IDP have been consistently variable in the literature. Early studies suggested a wide‐ranging 15%–37% risk of upgrade to atypia/HRL, and as much as 15%–17% risk of histologic upgrade to malignancy.^17^, ^18^, ^19^ Our own institution published a series in 2009 which identified an overall upgrade rate of 16.4%, found among 104 distinct cases of IDP that were later surgically excised. The study identified a 7.7% upgrade to ADH, and a considerable 8.7% upgrade to either in‐situ or invasive malignant disease. At the time, excision was recommended by the authors of the study, and the importance of radiologic‐pathologic correlation was highlighted.^20^ More recent studies, however, have seen a decrease in the predicted risk of upgrade. In a study published in 2018, Kiran et al.^21^ found a low overall upgrade risk of 7.3% in 153 patients: 1.3% for invasive cancer, 2.7% for DCIS and 3.3% for ADH. In 2019, Chen et al. reported an overall upgrade risk of 14.6% among 296 IDP (in 278 patients), with a 3.9% upgrade risk of DCIS and no invasive cancer.^22^ Our data reveal comparable numbers in all categories (13.4% of the overall patients, 12.3% to atypia/HRL and 1.1% to any malignancy). Of note, this study did include lobular neoplasia (atypical lobular hyperplasia/lobular carcinoma in situ) in the high‐risk category, which increased the number of upgrades compared to only considering ADH.

While identifying DCIS or invasive cancer brings about obvious clinical implications in terms of both local and systemic treatment, the clinical significance of identifying atypical or high‐risk cells in a papilloma is unclear, especially in patients with prior history of breast disease. The same study by Chen et al.^22^ looked at the implications of patient risk status, prior history of cancer or atypia and breast density when considering IDP upgrade on surgical excision. In it, the authors argue that high‐risk patients, defined as those with a >20% lifetime risk of developing breast cancer as per risk model calculations, are 2.5 times more likely to experience histologic upgrade of surgically excised IDP. Additionally, they found that patients with prior or concurrent history of atypia or cancer have a statistically significant increase in risk for upgrade and are 2.7 times more likely to show upgrade. Lastly, they examined breast density as a marker of increased risk and found that it was not a significant predictor of upgrade.

Similarly, our study found that a patient's prior history of breast carcinoma was one of the few significant predictors for pathologic upgrade. Interestingly, among the 40 patients with a personal history of breast cancer in the entire cohort, 32.5% ended up upgrading on final excision (27.5% to atypia/HRL and 5% to a new carcinoma). This differs for patients with no prior history of high‐risk or malignant disease, whose upgrade rate was lower overall 11.2%, and furthermore only 0.8% of these upgraded to cancer. Our data supports the notion that a patient's previous history of breast cancer should be considered when evaluating the need for surgical excision of IDP diagnosed on CNB.

This study was retrospective, which presents well known biases in the abstraction of data from available electronic medical records. Additionally, our work stems from a breast cancer center, where a high overall volume of malignant disease is treated and where routine imaging follow up is the standard. To our understanding, at 490 discrete IDP (in 439 patients), this is one of the largest series to examine pure IDP on CNB followed by surgical excision—but even then, the study is still limited by stemming from a single academic institution.

In conclusion, management of pure IDP without atypia continues to vary among institutions and practicing breast surgeons, even within the same institutions. Our data do suggest a low upgrade to malignancy, 1.1% in this large patient cohort, and even then, mostly DCIS. Meanwhile, 12.3% of patients were upgraded to atypia or other HRL—patients that could benefit from changes in risk‐reducing clinical/radiologic management and follow‐up. The clinical significance of identifying atypia in a papilloma remains unknown, especially in a patient with a prior history of atypia, who may already be followed more closely. However, when we looked at prior history of breast disease, the majority of patients who were upgraded to either atypia or cancer had none, and would therefore be considered true clinical upgrades. As such, we conclude that excision for pure IDP after CNB without atypia should be considered if an upgrade to atypia would be of clinical value in management, and in patients with a prior history of malignancy where upgrade rate to a second malignancy was higher.

## CONFLICT OF INTEREST

Authors report no conflicts of interest.

## ETHICAL STATEMENT

This retrospective study was approved by our Institutional Review Board (IRB) and met all guidelines for such studies. The manuscript is not published elsewhere.

## AUTHOR CONTRIBUTIONS

All authors had full access to the data in the study and take responsibility for the integrity of the data and the accuracy of the data analysis. *Conceptualization*, R.S., S.J.; *Methodology*, R.S., E.M., M.R.; *Formal Analysis*, M.R.; *Resources*, K.P.; *Writing ‐ Review & Editing*, S.J.; *Visualization*, R.S.; *Supervision*, K.P., S.J.; *Software*, E.M., M.R.; *Validation*, E.M.; *Data Curation*, S.W., A.S., K.P.; *Project Administration*, S.J.

## Data Availability

The data that support the findings of this study are available from the corresponding author upon reasonable request.
